# Clinical practice guideline for female fertility preservation

**DOI:** 10.3389/fmed.2025.1730617

**Published:** 2026-02-02

**Authors:** Ningxia Sun, Haixia Ding, Lingbo Cai, Ri-Cheng Chian, Xiaohui Deng, Yichun Guan, Lei Jin, Xiaolin La, Ge Lin, Xiufeng Ling, Zhijuan Lou, Weiying Lu, Qun Lv, Fei Ma, Xiuying Pei, Song Quan, Jianzhen Shen, Minfang Tao, Lei Wang, Xiuxia Wang, Guangwu Xiong, Jian Xu, Peng Xu, Weihai Xu, Yuanqing Yao, Xiaoling Yuan, Fuqing Zhang, Qinhua Zhang, Qingxue Zhang, Xuehong Zhang, Ping Zhou, Wen Li

**Affiliations:** 1 Department of Reproductive Medicine, Second Affiliated Hospital of Naval Medical University, Shanghai, China; 2 Center for Reproductive Medicine & Fertility Preservation Program, International Peace Maternity and Child Health Hospital, School of Medicine, Shanghai Jiao Tong University, Shanghai, China; 3 Shanghai Key Laboratory of Embryo Original Disease, Shanghai, China; 4 The First Affiliated Hospital with Nanjing Medical University, Nanjing, China; 5 Laboratory of Research and Development, ARSCI Biomedical Inc., Jiaxing, China; 6 Center for Reproductive Medicine, Department of Obstetrics and Gynecology, Qilu Hospital of Shandong University, Jinan, China; 7 The Third Affiliated Hospital of Zhengzhou University, Zhengzhou, China; 8 Reproductive Medicine Center, Tongji Hospital, Tongji Medicine College, Huazhong University of Science and Technology, Wuhan, China; 9 The First Affiliated Hospital of Xinjiang Medical University, Urumqi, China; 10 Reproductive and Genetic Hospital of CITIC-Xiangya, Changsha, China; 11 Women’s Hospital of Nanjing Medical University, Nanjing Women and Children’s Healthcare Hospital, Nanjing, China; 12 Ruikang Hospital Affiliated to Guangxi University of Chinese Medicine, Nanning, China; 13 Hainan Women and Children’s Medical Center, Haikou, China; 14 Sichuan Provincial People’s Hospital, School of Medicine, University of Electronic Science and Technology of China, Chengdu, China; 15 Department of Medical Oncology, National Cancer Center/Cancer Hospital, Chinese Academy of Medical Sciences and Peking Union Medical College, Beijing, China; 16 Key Laboratory of Fertility Preservation and Maintenance of Ministry of Education, School of Basic Medical Science, Ningxia Medical University, Yinchuan, Ningxia, China; 17 Nanfang Hospital, Southern Medical University, Guangzhou, China; 18 Fujian Medical University Union Hospital, Fuzhou, Fujian, China; 19 Department of Gynecology and Obstetrics, Shanghai Sixth People’s Hospital Affiliated to Shanghai Jiao Tong University School of Medicine, Shanghai, China; 20 Department of Reproductive Center, Dalian Women and Children’s Medical Group, Dalian, China; 21 Center of Reproductive Medicine, Shengjing Hospital of China Medical University, Shenyang, Liaoning, China; 22 Shenyang Reproductive Health Clinical Medicine Research Center, Shenyang, Liaoning, China; 23 Department of Obstetrics and Gynecology, Beijing Tsinghua Changgung Hospital, School of Clinical Medicine, Tsinghua Medicine, Tsinghua University, Beijing, China; 24 Department of Obstetrics and Gynecology, Center for Reproductive Medicine, The Fourth Affiliated Hospital of School of Medicine, Zhejiang University, Yiwu, China; 25 Hainan JingHua Fertility and Obstetrics Hospital, Haikou, China; 26 The First Affiliated Hospital, Zhejiang University School of Medicine, Hangzhou, China; 27 Department of Obstetrics and Gynecology, The General Hospital of Chinese PLA, Beijing, China; 28 School of Nursing, Shanghai Jiao Tong University, Shanghai, China; 29 Women & Infants Hospital of Zhengzhou, Zhengzhou, China; 30 Shuguang Hospital, Shanghai University of Traditional Chinese Medicine, Shanghai, China; 31 Sun Yat-sen Memorial Hospital, Sun Yat-sen University, Guangzhou, China; 32 The First Affiliated Hospital of Lanzhou University, Lanzhou, China, China; 33 The First Affiliated Hospital of Anhui Medical University, Hefei, China

**Keywords:** clinical guideline, fertility assessment, fertility preservation, oocyte cryopreservation, ovarian stimulation, ovarian tissue cryopreservation

## Abstract

**Introduction:**

Female fertility preservation (FFP) has become a clinical priority because gonadotoxic therapies for cancer and benign diseases are increasingly common and may cause irreversible ovarian failure. The current clinical practice guideline provides evidence-based recommendations on fertility assessment, oocyte/embryo cryopreservation, and ovarian tissue cryopreservation and transplantation for women at risk of iatrogenic infertility.

**Methods:**

This guideline was developed in accordance with the WHO Handbook for Guideline Development. A multidisciplinary Guideline Development Group (GDG) formulated nine key clinical questions in the field of FFP, and Cochrane-standard systematic reviews were conducted for each question. The certainty of the evidence was assessed using the GRADE approach, with critical outcomes including live birth, clinical pregnancy, time to pregnancy, treatment-related delays in oncotherapy, and severe ovarian hyperstimulation syndrome (OHSS). Recommendations were formulated by the GDG through the GRADE Evidence-to-Decision framework.

**Results and discussion:**

The GDG agreed on nine recommendations tailored to the Chinese clinical practice environment. When cancer treatment must start within two weeks, a random-start stimulation protocol is conditionally endorsed; pooled data show only one extra mature oocyte, but the time saved outweighs the marginal gonadotrophin increase. Letrozole co-administration is strongly recommended because it restrains oestradiol without reducing yield and may lessen OHSS risk in hormone-sensitive tumours. For ovarian tissue cryopreservation, slow freezing and vitrification are deemed equivalent in the absence of comparative trials; institutional capacity dictates the choice. Concurrent GnRH-agonist during chemotherapy is strongly advised across seventeen RCTs and improves subsequent live birth. Oocyte cryopreservation is weakly preferred to tissue for sexually mature women on the basis of higher cumulative pregnancy and lower morbidity, while ovarian tissue cryopreservation remains the default when stimulation is impossible. Baseline fertility evaluation should combine age, AMH and AFC; no single marker is superior, yet together they refine counselling. Orthotopic transplantation is strongly favoured over heterotopic grafting because published live births are almost exclusively pelvic. Finally, ovarian cortical fragments should undergo routine histopathology, augmented—according to metastatic risk—by immunohistochemistry, PCR or murine xenograft; tissue harbouring malignant cells is usually withheld from re-implantation.

## 1 Background

Fertility preservation for women—especially those facing cancer treatments, severe autoimmune diseases, hematopoietic stem-cell transplantation, or premature ovarian insufficiency—has gained widespread attention in recent years (1). Cancer incidence rates are rising annually, with a trend toward younger ages at onset, while advancements in cancer treatment are improving long-term survival for patients (2). Benign hematological diseases in children, the broad application of hematopoietic stem cell and bone marrow transplantation, and chemotherapy for autoimmune diseases are also causing varying degrees of irreversible damage to female reproductive function (3, 4). Fertility preservation has become particularly important to improve the quality of life for these long-term survivors.

Various cancer and fertility preservation associations, such as the American Society for Reproductive Medicine (ASRM), the International Society of Fertility Preservation (ISFP), and the European Society of Human Reproduction and Embryology (ESHRE), have successively developed relevant guidelines and expert consensus documents (1, 5, 6). In 2021, the Fertility Preservation Committee of the Chinese Maternal and Child Health Association also issued the *Chinese Expert Consensus on Clinical Practice for Female Fertility Preservation* (7).

Most existing guidelines and expert consensus documents are based on expert group clinical experience and meeting discussions, which lead to consensus-based conclusions but lack strong evidence-based support. To guide the scientific and standardized implementation of fertility preservation technologies and to provide scientifically reliable supporting evidence, the Fertility Preservation Committee of the Chinese Maternal and Child Health Association initiated the development of this clinical practice guideline. This guideline provides recommendations for current issues in fertility preservation, serving as practical guidance for the standardized implementation and promotion of fertility preservation technologies.

## 2 Target users and target population

The target guideline users are physicians, including reproductive medicine specialist, gynaecologists, family physicians; nurses, including registered nurses and nurse practitioners; medical trainees, including medical students, residents, and fellows; and all other health care providers. The target population of this guideline are women with cancer undergoing fertility preservation.

## 3 Guideline methodology

The methodology for developing this guideline strictly follows the international process for evidence-based practice guidelines, using the Grading of Recommendations, Assessment, Development, and Evaluations (GRADE) system to evaluate the certainty of evidence and classify recommendation strength. This guideline is registered with the Guidelines International Network.^1^

### 3.1 Composition of the guideline panel

The guideline development group (GDG) members consists of reproductive medicine and obstetrics/gynecology specialists from across mainland China.

### 3.2 Identification of clinical questions

A questionnaire was distributed to all GDG members to collect clinical questions of high relevance to female fertility preservation. Following multiple rounds of discussion and revision by the clinical chair and methodology chair, nine clinical questions were identified for inclusion in the guideline. For each clinical question, the GDG prioritized outcomes based on clinical importance. Key outcomes of interest included post-preservation pregnancy and live birth rates, cancer recurrence and survival for cancer patients, and neonatal health.

### 3.3 Literature search and evidence synthesis

For each clinical question, evidence synthesis was conducted in line with the systematic review process of the Cochrane Collaboration.

A librarian developed comprehensive search strategy for each clinical question, using the PubMed, Embase, Web of Science, Cochrane Library, CNKI, Wanfang Data, CBM, and VIP databases. The full search strategy is available in Appendix. Searches covered the period from database inception to August 9, 2023. Additionally, studies included in relevant systematic reviews were also screened for inclusion, and GDG members were consulted to identify additional relevant studies.

For each clinical question, inclusion and exclusion criteria were defined prior to conducting the literature search. Standardized data extraction forms were then developed in Excel. Randomized controlled trials (RCTs) were prioritized, supplemented by observational studies where RCT data were insufficient. Two independent reviewers conducted literature screening and data extraction, with disagreements resolved through discussion with a third reviewer. The quality of the included RCTs was assessed using the Cochrane risk of bias tool (8).

For controlled studies with normally distributed data, analysis was conducted using RevMan 5.3 with a fixed-effects model for meta-analysis, with RCT data and observational data analyzed separately. For binary outcomes we calculated relative risk (RR) with 95% confidence intervals (CIs), while continuous outcomes we calculated mean difference (MD) with 95% CIs. Non-controlled study data were analyzed using R version 4.1.1. Heterogeneity of the meta-analysis was assessed using chi-square tests and *I*^2^ statistics, with *p* < 0.1 and *I*^2^ > 50% indicating heterogeneity. Where meta-analysis was not feasible, results were summarized descriptively in tables or text.

### 3.4 Certainty assessment of evidence

The GRADE method was used to evaluate the certainty of evidence and the GDG referenced these certainty assessments when formulating the recommendations. GRADE proceeds in two steps: first, the evidence certainty for each clinical outcome is rated—starting at high for RCTs or low for observational studies—and then downgraded (or, exceptionally, upgraded) for risk of bias, inconsistency, indirectness, imprecision, publication bias, large effect, plausible confounding and dose response gradient. The certainty of evidence was graded as A (high), B (moderate), C (low), or D (very low), reflecting progressively less confidence that the true effect lies close to the pooled estimate. The summary of the evidence is presented in the summary-of-findings (SoF) tables in Appendix, which list the relative and absolute effect estimates for each outcome along with the certainty of the evidence.

### 3.5 Formulation of recommendations

We structured the entire decision process with the GRADE Evidence-to-Decision (EtD) framework (9). This tool makes the judgements explicit by integrating benefits, harms, evidence certainty, patient values, resource use, cost-effectiveness, equity, and feasibility; the resulting recommendations are graded as either strong (clear net benefit/harm and high confidence) or conditional (uncertain balance or lower confidence) (10). The GDG members met by video conference on December 2023. Together with the methodologist and systematic-review team they reviewed the graded evidence summaries, discussed each EtD domain, and reached consensus on both the direction and strength of every recommendation (Table 1).

**Table 1:** Summary of recommendations.

Recommendations	Strength of evidence and recommendation
The GDG suggests the use of a random-start protocol for women with cancer urgently needing fertility preservation, especially those requiring cancer treatment within 2 weeks and therefore at risk of impaired fertility	Conditional recommendation ⨁◯◯◯
2. The GDG recommends adding letrozole to the ovarian stimulation protocol for women with cancer undergoing fertility preservation	Strong recommendation ⨁◯◯◯
3. The GDG suggests that fertility preservation institutions select appropriate methods of ovarian tissue cryopreservation (either slow freezing or vitrification) based on their own conditions and the specific needs of patients.	No recommendation ⨁◯◯◯
4. The GDG recommends the use of GnRH agonists for ovarian protection in women with autoimmune diseases or cancer undergoing chemotherapy	Strong recommendation ⨁⨁⨁◯
5. The GDG suggests oocyte cryopreservation for sexually mature female cancer patients requiring fertility preservation	Weak recommendation ⨁⨁◯◯
6. The GDG recommends a comprehensive evaluation of the patient’s age, as well as indicators such as AMH level and AFC, to assess their fertility status	Strong recommendation ⨁⨁◯◯
7. The GDG recommends orthotopic transplantation for women undergoing fertility preservation with OTC	Strong recommendation ⨁◯◯◯
8. The GDG suggests that for women with cancer planning to undergo fertility preservation, pathological examination should be used to assess the presence of tumors in ovarian tissue. For female cancer patients with an intermediate risk of ovarian metastasis, a combination of pathological examination, immunohistochemistry, and PCR should be employed for detection. For female cancer patients with a high risk of ovarian metastasis, ovarian transplantation is generally not recommended. If exceptional circumstances necessitate transplantation, it should be considered cautiously only after multiple detection methods, including pathological examination, immunohistochemistry, PCR, and xenotransplantation into nude mice, have yielded negative results	Conditional recommendation ⨁◯◯◯
9. The GDG suggests that for post pubertal patients with DOR undergoing fertility preservation, OC should be considered. For prepubertal patients with DOR, such as those with Turner syndrome, OTC should be considered	Conditional recommendation ⨁◯◯◯

D-level evidence = ⨁◯◯◯; C-level evidence = ⨁⨁◯◯; B-level evidence = ⨁⨁⨁◯.

## 4 Recommendations

### 4.1 Clinical question 1: For women with cancer undergoing fertility preservation, should a random-start protocol or a conventional start protocol be selected for controlled ovarian stimulation?

#### 4.1.1 Recommendation

The GDG suggests the use of a random-start protocol for women with cancer urgently needing fertility preservation, especially those requiring cancer treatment within 2 weeks and therefore at risk of impaired fertility (conditional recommendation, D-level evidence).

#### 4.1.2 Implementation recommendations

For women with cancer requiring oocyte cryopreservation (OC), if fertility preservation must be completed within 2 weeks, the random-start protocol for ovarian stimulation should be appropriately selected based on the patient’s primary disease, ovarian function, and follicle development. If cancer treatment can be delayed, either a conventional or random-start protocol may be selected.

#### 4.1.3 Summary of evidence

Four systematic reviews (11–14) and 31 observational studies (15–45) were included in this analysis, with the primary focus on patients with breast cancer, whose ages ranged from 13 to 44 years. The baseline antral follicle count (AFC) of the patients ranged from 6 to 22, and the mean baseline anti-Müllerian hormone (AMH) level was approximately 2–3 ng/mL. The ovarian stimulation protocols varied across the studies, with the total amount of gonadotropins administered ranging from about 1,500 to 5,500 IU. The protocol duration varied from as short as 5 days to as long as 18 days.

Meta-analysis results suggested that, compared with the conventional start protocol, the random-start protocol may slightly improve the numbers of retrieved oocytes [MD = 0.56 (95% CI, 0.05, 1.08); D-level evidence] and metaphase II (MII) oocytes [MD = 1.25 (95% CI, 0.80, 1.70); D-level evidence] in women with cancer. It also appeared to improve fertilization rates [MD = 9.38 (95% CI, 5.96, 12.81); D-level evidence] and two pronuclei (2PN) rates [MD = 11.83 (95% CI, 8.01, 15.65); D-level evidence]. The random-start protocol may not affect the number of available embryos [MD = 0.10 (95% CI, −0.60, 0.80); D-level evidence] but may increase the rate of high-quality embryos [MD = 1.36 (95% CI, −2.95, 5.67); D-level evidence]. However, the confidence in this body of evidence was low, and high-quality clinical trial results are needed to validate the current effect estimates.

Regarding the safety of the stimulation protocols, two studies (29, 37) reported moderate to severe ovarian hyperstimulation syndrome (OHSS), with incidence rates of 9 and 1.5%. In addition, meta-analysis results suggested that the random-start protocol may require a higher total dose of gonadotropins than the conventional start protocol [MD = 187.17 IU (95% CI, 114.60, 259.73); D-level evidence], but may not have a significant impact on the duration of stimulation [MD = 0.75 days (95% CI, 0.56, 0.93); D-level evidence].

#### 4.1.4 Rationale for recommendation

Current evidence shows a lack of high-quality RCTs comparing the effects of random-start and conventional start protocols. However, multiple observational studies suggest that random-start protocols may offer benefits over the conventional start protocol, although our confidence in the magnitude of this effect is not high. Based on the study data and experience of the GDG members, it appears that the total dose of gonadotropins required for random-start protocols may be higher than for conventional start protocols, while no significant differences have been observed in the duration of ovarian stimulation. No data were found to indicate differences in cost-effectiveness or variation in resource consumption between the two protocols. The random-start protocol is widely adopted in fertility preservation, both in China and internationally. Patient and provider acceptance of this protocol is comparable to that of conventional protocols. Its recommendation would not be expected to adversely affect equity in fertility preservation services for cancer patients.

### 4.2 Clinical question 2: For women with cancer undergoing fertility preservation, is adding letrozole to the ovarian stimulation protocol recommended?

#### 4.2.1 Recommendation

The GDG recommends adding letrozole to the ovarian stimulation protocol for women with cancer undergoing fertility preservation (strong recommendation, D-level evidence).

#### 4.2.2 Implementation suggestions

For patients with hormone-sensitive cancers (such as breast cancer), the addition of letrozole is recommended. For patients with a high ovarian response, letrozole is recommended to reduce the risk of OHSS. For non-hormone-sensitive cancers, adding letrozole can be considered based on the patient’s circumstances.

#### 4.2.3 Summary of evidence

Three systematic reviews (13, 46, 47) and nine observational studies (19, 31, 48–54) were included in this analysis, which primarily focused on patients with breast cancer or those undergoing gonadotoxic treatment. The average baseline AFC of the patients ranged from 10.0 to 21.1, and the average baseline AMH level ranged from 2.0 to 4.1 ng/mL. The dose of letrozole added to the ovarian stimulation protocols ranged from 2.5 to 5.0 mg per day.

The results of meta-analysis showed that, compared with ovarian stimulation protocols without letrozole, adding letrozole may not affect the number of retrieved oocytes [MD = −0.47 (95% CI, −1.45, 0.51); D-level evidence] or MII oocytes [MD = −0.10 (95% CI, −1.01, 0.81); D-level evidence]. Additionally, protocols with letrozole may require a higher total dose of gonadotropins [MD = 86.74 IU (95% CI, −55.24, 228.72); D-level evidence], but there was no difference in the duration of ovarian stimulation [MD = 0.11 days (95% CI, −0.13, 0.35); D-level evidence].

#### 4.2.4 Rationale for recommendation

While current evidence indicates that adding letrozole may not markedly enhance ovarian stimulation outcomes in cancer patients, the GDG, drawing on their clinical experience, concluded that it can reduce the risk of OHSS and improve endometrial receptivity. In patients with hormone-dependent tumors, such as breast cancer, the addition of letrozole during ovarian stimulation can reduce serum estrogen levels, which plays an important role in controlling disease progression, reducing the risk of recurrence. Therefore, the benefits of letrozole were considered to outweigh the potential risks. Additionally, the inclusion of letrozole in ovarian stimulation protocols is widely accessible and does not incur additional medical costs for patients. For these reasons, the expert group recommended adding letrozole to the stimulation protocols of cancer patients undergoing fertility preservation.

### 4.3 Clinical question 3: Compared with slow freezing, is vitrification recommended for ovarian tissue cryopreservation (OTC)?

#### 4.3.1 Recommendation

The GDG suggests that fertility preservation institutions select appropriate methods of OTC (either slow freezing or vitrification) based on their available resources and the specific needs of patients (no-preference recommendation; D-level evidence).

#### 4.3.2 Implementation suggestions

Institutions that perform slow freezing of ovarian tissue should conduct a thorough assessment of their freezing equipment and facilities, and operators must undergo rigorous training. For institutions without the capacity for slow freezing, vitrification should be performed by operators who have received specialized training in OTC for fertility preservation.

#### 4.3.3 Summary of evidence

Four systematic reviews (55–58) of *in vitro* studies were included, with human ovarian tissue as the study samples. Compared with slow freezing, vitrification was found to improve several indirect clinical indicators: higher proportions of intact primordial follicles and normal stromal cells, a greater rate of morphologically normal primordial follicles, and a lower DNA fragmentation rate. Additionally, 21 observational studies (59–79) were included, with sample size ranging from 2 to 1,810 and ages ranging from 2 to 45 years.

In the absence of controlled studies, the systematic-review team separately summarized clinical outcomes for the slow-freezing and vitrification groups. Meta-analysis results indicated that the pregnancy rate in the slow-freezing group was estimated to be 0.38 (95% CI, 0.33, 0.44; D-level evidence), and the live birth rate was 0.31 (95% CI, 0.26, 0.36; D-level evidence). In the vitrification group, the pregnancy rate was 0.10 (95% CI, 0.04, 0.23; D-level evidence), and the live birth rate was also 0.10 (95% CI, 0.03, 0.25; D-level evidence). Additionally, the miscarriage rate among patients undergoing slow freezing was 0.14 (95% CI, 0.08, 0.23; D-level evidence), compared with 0.26 (95% CI, 0.05, 0.70, D-level evidence) in those undergoing vitrification. The rate of menstrual recovery after transplantation in the slow-freezing group was 0.91 (95% CI, 0.84, 0.95, D-level evidence), with a recovery time of approximately 3 to 4 months [mean = 3.68 (95% CI, 3.41, 3.96); D-level evidence]. No studies were found reporting menstrual recovery following transplantation in patients undergoing vitrification of ovarian tissue.

#### 4.3.4 Rationale for recommendation

Currently, the vast majority of clinical pregnancy reports following ovarian tissue transplantation are based on the use of slow freezing. Due to its ease of operation and lack of need for large-scale equipment, vitrification has been widely adopted by most fertility preservation institutions, as it shows no significant differences from slow freezing in morphological indicators. However, its safety still requires confirmation through high-quality clinical studies. Compared with vitrification, slow freezing of ovarian tissue requires more sophisticated equipment, facilities, and personnel, and involves greater consumption of medical resources and costs. Therefore, after comprehensive consideration, the expert panel made a no-preference recommendation on this issue.

### 4.4 Clinical question 4: For patients undergoing chemotherapy, should the use of GnRH agonists be recommended for ovarian protection?

#### 4.4.1 Recommendation

The GDG recommends the use of GnRH agonists for ovarian protection in women with autoimmune diseases or cancer undergoing chemotherapy (strong recommendation, B-level evidence).

#### 4.4.2 Implementation suggestions

For women undergoing chemotherapy, administration of a long-acting formulation of GnRH agonists (GnRH-a) every 4 weeks is recommended. Alternatively, clinicians may select the formulation and dosage based on the patient’s treatment schedule and disease condition.

#### 4.4.3 Summary of evidence

Seventeen RCTs (80–96) were included in this analysis, with most study populations comprising patients with malignancies (including breast cancer, lymphoma, cervical cancer, and ovarian cancer). One study focused on patients with systemic lupus erythematosus. All trials compared the efficacy and safety of administering GnRH-a before or during chemotherapy versus not administering GnRH-a. The primary GnRH-a agents used were triptorelin and goserelin.

Compared with no GnRH-a administration, meta-analysis showed that using GnRH-a before and during chemotherapy significantly reduced the incidence of ovarian failure [RR = 0.40 (95% CI, 0.30, 0.53); a reduction of 179 cases per 1,000 individuals (95% CI, reduction of 209 to 140 cases); B-level evidence]. One study reported that administering GnRH-a before and during chemotherapy significantly increased AFC [MD = 3.02 (95% CI, 1.08, 4.96); D-level evidence]. Another study found no significant effect on AMH levels [MD = −0.57 (95% CI, −8.22, 7.08); D-level evidence]. Moreover, meta-analysis showed that using GnRH-a before and during chemotherapy significantly reduced follicle-stimulating hormone (FSH) levels (IU/L) [MD = −12.09 (95% CI, −13.02, −11.16); D-level evidence] while also increasing pregnancy rates [RD = 0.10 (95% CI, 0.05, 0.14); B-level evidence] and live birth rates [RD = 0.21 (95% CI, 0.13, 0.29); C-level evidence].

#### 4.4.4 Rationale for recommendation

Current research findings indicate that administering GnRH-a before and during chemotherapy has no significant effect on AMH levels but may reduce the incidence of premature ovarian failure, increase the number of antral follicles, decrease FSH levels, and improve pregnancy and live birth rates. Given that the outcomes associated with GnRH-a administration were not inferior to those in the non-administration group—and that the non-administration group may experience severe ovarian dysfunction due to chemotherapy—the expert panel, after fully considering the feasibility, acceptability, and impact on health service equity, recommended the use of GnRH-a for ovarian protection in women undergoing chemotherapy.

### 4.5 Clinical question 5: For sexually mature female cancer patients undergoing fertility preservation, is OC or OTC recommended?

#### 4.5.1 Recommendation

The GDG suggests OC for sexually mature female cancer patients requiring fertility preservation (weak recommendation, C-level evidence).

#### 4.5.2 Implementation suggestions

For patients urgently requiring cancer treatment, ovarian stimulation may not be feasible; therefore, OTC is recommended for fertility preservation. In cases of hormone-sensitive tumors, such as certain types of breast cancer, the potential impact of ovarian stimulation on the primary disease and the benefits of fertility preservation should be carefully weighed, and the preservation method selected with caution.

#### 4.5.3 Summary of evidence

Forty-six observational studies (31, 60, 61, 63, 64, 66–69, 73, 76, 78, 97–130) were included in this analysis, with study populations consisting exclusively of cancer patients whose mean ages ranged from 15 to 40 years.

In the absence of controlled studies, the systematic review team separately summarized clinical outcomes for the OC and OTC groups. Meta-analysis results indicated that the pregnancy rate in the OC group was 0.43 (95% CI, 0.35, 0.51; D-level evidence), with a live birth rate of 0.33 (95% CI, 0.27, 0.41; D-level evidence). In contrast, the pregnancy rate in the OTC group was 0.35 (95% CI, 0.32, 0.38; D-level evidence), and the live birth rate was 0.27 (95% CI, 0.25, 0.30; D-level evidence). The miscarriage rate in the OC group was higher than that in the OTC group (0.50 vs. 0.30). Additionally, tumor recurrence rates were low in both groups (0.09 vs. 0.08), while the tumor-related mortality rate was 0.06 (95% CI, 0.05, 0.08) in the OC group (D-level evidence) and 0.11 (95% CI, 0.09, 0.13) in the OTC group (D-level evidence).

#### 4.5.4 Rationale for recommendation

For prepubertal females, OTC is the only available option for fertility preservation. For postpubertal female cancer patients, the choice of fertility preservation technique should be made by clinicians in consideration of the patient’s primary disease. The evidence indicates that OC is associated with higher pregnancy and live birth rates compared with OTC, while miscarriage rates, tumor recurrence rates, and tumor-specific survival rates are comparable between the two methods. Additionally, clinical data on OTC are limited by small sample sizes and low-quality evidence. Given that OC is a more established technique, involving less procedural trauma and greater acceptability, the GDG suggests OC for fertility preservation in postpubertal female cancer patients. However, given the low quality of the evidence base, future research findings may change the current conclusions; therefore, the GDG made only a weak recommendation. The fertility-preservation plan must be tailored after weighing the patient’s preferences, the urgency of cancer treatment, and the biological features of the primary disease.

### 4.6 Clinical question 6: For women with malignant tumors planning to undergo ovarian stimulation, what methods can be used to assess their fertility status?

#### 4.6.1 Recommendation

The GDG recommends a comprehensive evaluation of the patient’s age, as well as indicators such as AMH level and AFC, to assess their fertility status (strong recommendation, C-level evidence).

#### 4.6.2 Implementation suggestions

For women with cancer requiring fertility assessment, baseline AFC can be evaluated via ultrasound during the early follicular phase, and AMH levels can be tested at any point in the menstrual cycle. After these assessments, clinicians should consider the patient’s age and other clinical factors to provide a comprehensive evaluation of fertility.

#### 4.6.3 Summary of evidence

Twelve observational studies (131–142) were included in this analysis, with the study populations comprising patients with breast cancer or lymphoma. The mean baseline body mass index of the patients ranged from 21.7 to 23.1 kg/m^2^, the mean baseline AFC ranged from 12.5 to 22.8, and the mean baseline AMH level ranged from 2.3 to 4.6 ng/mL.

Two studies (139, 141) indicated that AMH and FSH levels in patients may predict the decline in ovarian function following chemotherapy. Four studies found that AMH level and AFC in cancer patients significantly influenced the number of mature oocytes retrieved. However, current studies reached inconsistent conclusions on whether AMH level and AFC can predict the total number of oocytes retrieved in patients with cancer. A study found that both AMH level and AFC were associated with the number of cumulus-oocyte complexes retrieved (133). Raad et al. (134) showed that, using univariate analysis with AMH = 1.5 or AFC = 12 as stratification thresholds, there was no significant difference in oocyte retrieval rates between the two groups.

In addition, clinical studies on non-cancer patients were considered as indirect evidence. AFC showed good sensitivity (0.72–0.94) but variable specificity (0.39–0.97) in predicting ovarian response to stimulation, and AMH level demonstrated similar sensitivity (0.63–1.00) and specificity (0.41–0.94). When AFC and AMH level were combined as composite indicators, sensitivity reached 0.74 and specificity was 0.72. In predicting ovarian reserve function, AFC had a sensitivity of 0.61–0.93 and a specificity of 0.76–0.99, while AMH level had a sensitivity of 0.66–0.98 and a specificity of 0.34–0.96.

#### 4.6.4 Rationale for recommendation

Fertility assessment is an important predictor of patient outcomes following ovarian stimulation. Currently, the most commonly used fertility assessment indicators are AFC and AMH level. However, high-quality studies comparing the predictive value of different assessment methods are lacking. Existing small-sample studies show inconsistent conclusions when using AFC or AMH level alone to assess outcomes of ovarian stimulation. In contrast, combining AFC and AMH level with other indicators has demonstrated higher sensitivity and specificity in evaluating ovarian function. Therefore, the expert panel recommended integrating AFC, AMH level, age, and other relevant indicators in female cancer patients undergoing ovarian stimulation.

### 4.7 Clinical question 7: For patients undergoing fertility preservation via OTC, should orthotopic or heterotopic transplantation be recommended?

#### 4.7.1 Recommendation

The GDG recommends orthotopic transplantation for patients undergoing fertility preservation with OTC (strong recommendation, D-level evidence).

#### 4.7.2 Implementation suggestions

For patients receiving OTC, orthotopic transplantation is recommended unless special circumstances apply. In cases where patients have undergone multiple pelvic surgeries with severe adhesions, received pelvic chemotherapy, or have other conditions that preclude orthotopic transplantation, heterotopic transplantation may be considered. Follicle development should be continuously monitored following heterotopic transplantation, and *in vitro* fertilization may be offered.

#### 4.7.3 Summary of evidence

Nineteen observational studies (62, 63, 74, 79, 98, 109, 116, 120, 130, 143–152) were included. Most study populations comprised women with cancer, with a small number of women having non-malignant diseases. The populations’ ages ranged from 21 to 42 years.

In the absence of controlled study results, the systematic review team separately summarized the outcome indicators for the orthotopic transplantation and heterotopic transplantation groups. Meta-analysis results indicated that the pregnancy rate in the orthotopic transplantation group was 0.34 (95% CI: 0.30–0.38; D-level evidence), with a live birth rate of 0.29 (95% CI: 0.25–0.33; D-level evidence). In the heterotopic transplantation group, the pregnancy rate was 0.38 (95% CI: 0.07–0.84; D-level evidence). The mean number of oocytes retrieved after orthotopic transplantation was higher than that after heterotopic transplantation (15.6 vs. 10.6), and the mean number of available embryos was also significantly greater (10.0 vs. 2.0).

#### 4.7.4 Rationale for recommendation

Thawed ovarian tissue can currently be transplanted either orthotopically (within the pelvic cavity) or heterotopically (outside the pelvic cavity). Orthotopic transplantation more closely replicates normal anatomical conditions and allows for the possibility of natural conception in some patients. In contrast, heterotopic transplantation is technically simpler and facilitates easier monitoring of subsequent follicle development. To date, few case reports have documented pregnancy or live birth rates following heterotopic transplantation, and most pregnancies have resulted from orthotopic transplantation. Given the limited data on heterotopic transplantation, orthotopic transplantation is preferred, due to its association with a higher number of retrieved oocytes and a greater number of available embryos. It is also generally more acceptable to patients during implementation. Therefore, the GDG recommended orthotopic transplantation. However, heterotopic transplantation may be considered in special circumstances where orthotopic transplantation is not feasible.

### 4.8 Clinical question 8: For patients planning to undergo fertility preservation, is it recommended to use a combination of multiple detection methods (immunohistochemistry, PCR, and xenotransplantation in nude mice), in addition to pathological examination, to assess tumor carriage in ovarian tissue?

#### 4.8.1 Recommendation

The GDG suggests that for women with cancer planning to undergo fertility preservation, pathological examination should be used to assess the presence of tumors in ovarian tissue. For female cancer patients with an intermediate risk of ovarian metastasis, a combination of pathological examination, immunohistochemistry, and PCR should be employed for detection. For female cancer patients with a high risk of ovarian metastasis, ovarian transplantation is generally not recommended. If exceptional circumstances necessitate transplantation, it should be considered cautiously only after multiple detection methods, including pathological examination, immunohistochemistry, PCR, and xenotransplantation into nude mice, have yielded negative results (conditional recommendation, D-level evidence).

#### 4.8.2 Implementation suggestions

For women with cancer planning to undergo OTC, pathological examination of the ovarian tissue should be routinely performed and meticulously documented at the time of cryopreservation. Additionally, the risk of ovarian metastasis should be assessed. For low-risk malignancies, pathological examination alone is sufficient. For intermediate-risk malignancies, a combination of pathological examination, immunohistochemistry, and PCR is recommended. For high-risk malignancies, OTC and transplantation are generally not recommended.

#### 4.8.3 Summary of evidence

Twenty-seven observational studies (153–179) were included, reporting outcomes of ovarian tissue tumor carriage assessment in women with cancer or hematological diseases prior to fertility preservation. Detection methods included PCR, immunohistochemistry, xenotransplantation in nude mice, and pathological examination. Sample sizes varied considerably, ranging from 5 to 735 cases. The mean age of women undergoing OTC was between 16.0 and 32.8 years, with the youngest patient aged 2 years and the oldest aged 41. The GDG summarized the detection rates of each method, revealing substantial variability across studies. The results of PCR detection were primarily influenced by the choice of target, with 14 studies (154, 156–158, 160, 162–164, 166, 169, 174, 175, 177, 179) reporting positive detection results and detection rates ranging from 9.09 to 75%. Immunohistochemistry yielded positive detection results in only two studies (160, 168), with detection rates of 2.13 and 9.09%. Xenotransplantation produced positive detection results in six studies (154, 158, 160–162, 175), with detection rates from 7.69 to 72.22%. Pathological examination yielded positive results in four studies (154, 162, 163, 172), with detection rates between 0.4 and 8.33%. Additionally, a limited number of studies reported follow-up results after ovarian tissue transplantation, with no cases of disease recurrence observed.

#### 4.8.4 Rationale for recommendation

The safety of ovarian transplantation in women with cancer is a critical concern for both clinicians and patients. Institutions that offer OTC typically rely on routine pathological examination to determine whether ovarian tissue has been infiltrated by tumor cells. However, pathological examination has relatively low sensitivity and specificity, resulting in suboptimal detection accuracy. Current research indicates that using immunohistochemistry, PCR, and xenotransplantation in nude mice to detect tumor cells in ovarian tissue yields varying positive detection rates. Nevertheless, due to the limited sample sizes and low quality of evidence in these studies, their findings cannot be generalized to represent the overall tumor infiltration status in all tissues. Considering both safety and clinical feasibility, the GDG recommended that patients planning to undergo fertility preservation routinely undergo pathological examination to assess tumor carriage in ovarian tissue, and that additional detection methods be applied based on the risk of ovarian metastasis.

### 4.9 Clinical question 9: For patients with diminished ovarian reserve (DOR) planning to undergo fertility preservation, should OC or OTC be recommended?

#### 4.9.1 Recommendation

The GDG suggests that for post pubertal patients with DOR undergoing fertility preservation, OC should be considered. For prepubertal patients with DOR, such as those with Turner syndrome, OTC should be considered (conditional recommendation, D-level evidence).

#### 4.9.2 Implementation suggestions

For postpubertal women with DOR, OC is recommended for fertility preservation. For prepubertal patients with DOR or those at high risk of developing it (e.g., Turner syndrome), OTC is recommended to preserve fertility. For women aged over 38 years with severe DOR (AMH <0.5 ng/mL), pregnancy should be attempted as soon as possible, and fertility preservation techniques are not recommended.

#### 4.9.3 Summary of evidence

Six observational studies (74, 180–184) were included. The study populations consisted of patients with DOR, ovarian insufficiency, or ovarian dysfunction. The sample sizes were small, ranging from 37 to 100 participants, and patient ages ranged from 2 to 38 years. Owing to insufficient data to conduct a meta-analysis, the systematic review team provided only a descriptive summary of the study results. A study published in 2019 reported that among 18 patients who underwent OC, seven achieved pregnancy (184). A study from Japan reported that among 60 patients who underwent OTC, only three achieved pregnancy (74).

#### 4.9.4 Rationale for recommendation

Despite growing attention to fertility preservation in patients with DOR, high-quality evidence remains scarce. The GDG provided recommendations for patients with DOR across different age groups based on clinical experience. Further investigation of this issue will require results from high-quality clinical studies.

In addition to the recommendations above, the GDG further deliberated and reached consensus on the two key issues below in the field of female fertility preservation.

### 4.10 Consensus 1: What are the upper and lower age limits for fertility preservation in women?

1. The 2020 European Society of Medical Oncology and European Society of Human Reproduction and Embryology (ESHRE) guidelines do not recommend fertility preservation for women over the age of 36 (5). The *2021 Chinese Expert Consensus on Fertility Preservation* sets an upper age limit of 35 years for OTC and transplantation, and 40 years for OC (7).
2. For women with breast cancer, the expert consensus (*Hunan Province Young Female Breast Cancer Patients Fertility Preservation Implementation Plan Expert Consensus 2018*) recommends an upper age limit of 40 years for fertility preservation and ovarian function protection (185).
3. For women with early-stage endometrial cancer, the 2019 expert consensus also recommends an upper age limit of 40 years for fertility preservation (186). If the patient has a strong desire for fertility, this can be moderately extended to 45 years after evaluation by a medical team.
4. For women with early-stage cervical cancer, the *China Expert Consensus on Fertility Preservation in Early-Stage Cervical Cancer* suggests an upper age limit of 45 years (187).
5. For women with early-stage epithelial ovarian cancer, malignant ovarian germ cell tumors, and early-stage sex cord-stromal tumors, the *China Expert Consensus on Fertility Preservation in Malignant Ovarian Tumors* (2022 edition) requires an age of under 40 years to be considered for fertility preservation (188).
6. The 2023 *Chinese Expert Consensus on Fertility Preservation in Lymphoma Patients* recommends fertility preservation only for patients with Hodgkin’s lymphoma and low-grade non-Hodgkin’s lymphoma patients who are under the age of 40 (189).

### 4.11 Consensus 2: Basic setup recommendations for institutions conducting fertility preservation

#### 4.11.1 Multidisciplinary team and professional requirements

1. Interdisciplinary medical team and collaboration: Women undergoing fertility-threatening treatments require a multidisciplinary medical team. This team may include oncologists, reproductive endocrinologists, urologists, and reproductive surgeons trained in fertility preservation techniques.
2. Mental health guidance: Fertility preservation programs should have timely access to trained mental health professionals to provide counseling and support patients through what is often a difficult decision-making process.
3. Genetic counseling: For conditions that may be hereditary, genetic counselors should be available to discuss the potential risk of transmitting the disease to offspring and to conduct genetic testing.
4. Provider selection for patient consultation: The choice of provider to discuss fertility preservation and family planning options with cancer patients and their families should depend more on the provider’s expertise, the patient’s condition, and the availability of local fertility specialists, rather than being limited to a specific discipline. Possible providers include pediatric oncologists, endocrinologists (including pediatric endocrinologists), fertility specialists, specialist nurses, or other relevant healthcare providers.

#### 4.11.2 Facility requirements

Clinics offering fertility preservation should have the necessary infrastructure to provide immediate ovarian stimulation. Fertility preservation programs should be integrated with experienced assisted reproductive technology programs that offer a full range of fertility preservation techniques, including embryo cryopreservation and OC. These programs should be available year-round, be able to quickly accommodate patients, provide counseling to prepubescent patients, and ideally offer procedures such as ovarian and testicular tissue cryopreservation.

#### 4.11.3 Medical costs

Financial consultation and funding: In urgent situations, a coordinator should be involved to reach an agreement on fundraising methods. Financial counseling is recommended for patients seeking fertility preservation services due to the high cost of these technologies and the lack of insurance coverage in many cases. Ideally, funding consultations and flexible strategies should be provided to help address cost-related issues.

## 5 Discussion

Over the past decade, fertility preservation has advanced rapidly as an emerging field within reproductive medicine. However, constrained by disparities in socioeconomic development, economic conditions, traditional cultures, and religious beliefs across different countries and regions, the global implementation of fertility preservation remains asynchronous, with significant controversies persisting around numerous key issues. Addressing 11 prevalent clinical controversies, this fertility preservation guideline was developed using a rigorous methodology to provide standardized recommendations for clinical practice. The evidence synthesis and expert consensus highlight the importance of individualized approaches to fertility preservation, particularly in the context of oncological and non-oncological conditions that may impair fertility.

The guideline provides specific recommendations on various clinical questions, including the choice of ovarian stimulation protocols, the use of letrozole in stimulation regimens, and the choice between slow freezing and vitrification for OTC. Notably, the evidence supports the use of randomized ovarian stimulation protocols in urgent cases, which can significantly reduce the time required for fertility preservation while maintaining clinical outcomes. Additionally, the use of letrozole is recommended for hormone-sensitive tumors, as it may mitigate the risk of disease progression and reduce the incidence of OHSS in high responders.

In addressing the choice between OC and OTC, the guideline emphasizes the importance of considering patient-specific factors, such as age, tumor type, and urgency of cancer treatment. For sexually mature females, OC is recommended due to its higher pregnancy and live birth rates, while OTC remains the only option for prepubertal girls.

Compared with previous guidelines, such as those from ESHRE and ASRM, this guideline provides more detailed assessments of clinical efficacy and safety, particularly in different patient populations. For example, the recommendation for GnRH-a use in chemotherapy patients is supported by evidence showing its benefits in reducing ovarian failure and improving pregnancy outcomes.

Developed in strict accordance with international standards, this guideline integrates comprehensive evidence and thorough discussions to provide recommendations tailored to the Chinese clinical context. The evidence analysis and recommendations also offer valuable insights for international practice. Future research should focus on addressing the gaps in evidence, particularly regarding economic benefits, patient preferences, and long-term outcomes, to further improve the clinical practice of fertility preservation.

Finally, the guideline development process revealed significant challenges in the field. Fertility preservation suffers from a paucity of large-scale clinical studies, with the current body of evidence predominantly derived from low-quality sources such as case reports and single-center experiences. This evidence gap likely reflects the highly heterogeneous global implementation of fertility preservation services. Concerted efforts are therefore imperative to expand access and standardize practices across broader geographic regions. We anticipate that as fertility preservation becomes more widely adopted worldwide, ongoing controversies will be resolved through higher-quality evidence. Consequently, we will continue to update the clinical recommendations in future iterations of this guideline as new data emerge.

Given the conditional nature of most recommendations in this guideline, which are inherently sensitive to individual patient values and contexts, the principle of Shared decision making (SDM) is paramount. SDM is a structured, two-way conversation in which clinicians share evidence-based information on benefits, harms, and uncertainties, and patients disclose their goals, priorities, and context; both parties then reach a mutually agreed-upon plan (190, 191). Fertility preservation decisions are deeply personal, involving trade-offs between timing, treatment efficacy, personal health risks, future family goals, and emotional well-being. Therefore, clinicians must engage patients in a collaborative process—clearly communicating the conditional recommendations, the underlying certainty of evidence, and the potential benefits and burdens of each option—to ensure that the chosen path aligns with the patient’s unique priorities, circumstances, and preferences.

## Data Availability

The original contributions presented in the study are included in the article/Supplementary material, further inquiries can be directed to the corresponding authors.

## Footnotes

1
https://guidelines.ebmportal.com
