# Supplementary material for: Clinical practice guideline for female fertility preservation
Source: Front Med (Lausanne). 2026 Feb 2;12:1730617. doi: 10.3389/fmed.2025.1730617 (PMC12907358; doi:10.3389/fmed.2025.1730617)
Supplement: Supplementary file 1 [file Data_Sheet_1.pdf]

Key topic / question	Present guideline (this work)	ASRM committee opinion*	ESHRE guideline*	ISFP consensus / update*
Overall approach	Evidence-based, graded recommendations; adapted to national context and health-care system	Narrative + evidence-informed recommendations	Formal evidence grading and good-practice points	Expert consensus with selected evidence grading
Target population	Adult and adolescent females receiving gonadotoxic therapy or gonadectomy	Similar	Similar	Similar, with emphasis on oncologic and benign indications
First-line fertility preservation (FP) options	Recommends embryo/oocyte cryopreservation as first-line when time and ovarian reserve allow	Same	Same	Same
Ovarian tissue cryopreservation (OTC)	Recommends OTC for patients who cannot undergo stimulation or are prepubertal; emphasizes centralized expertise and long-term storage management	Recognized; still cautious in some indications	Recognized as established in experienced centers	Highlighted as key option, especially for young patients
Use of GnRHa for ovarian protection	Allows GnRHa as adjunctive option in selected indications but not as a replacement for FP	Similar cautious stance	Similar cautious stance	Similar cautious stance
Prepubertal girls	Supports OTC as the only feasible FP option, with strict ethical and multidisciplinary oversight	Similar	Similar	Similar
Experimental / emerging techniques (IVM, ART add-ons, etc.)	Considers these experimental and recommends use only in research settings with informed consent	Similar	Similar	Similar
